# Artificial intelligence-powered spatial analysis of tumor-infiltrating lymphocytes for prediction of prognosis in resected colon cancer

**DOI:** 10.1038/s41698-023-00470-0

**Published:** 2023-11-20

**Authors:** Yoojoo Lim, Songji Choi, Hyeon Jeong Oh, Chanyoung Kim, Sanghoon Song, Sukjun Kim, Heon Song, Seonwook Park, Ji-Won Kim, Jin Won Kim, Jee Hyun Kim, Minsu Kang, Sung-Bum Kang, Duck-Woo Kim, Heung-Kwon Oh, Hye Seung Lee, Keun-Wook Lee

**Affiliations:** 1 Lunit, Seoul, Republic of Korea; 2 Department of Internal Medicine, Seoul National University Bundang Hospital, Seoul National University College of Medicine, Seongnam, Republic of Korea; 3 Department of Pathology, Seoul National University Bundang Hospital, Seongnam, Republic of Korea; 4 Department of Surgery, Seoul National University Bundang Hospital, Seoul National University College of Medicine, Seongnam, Korea; 5 Department of Pathology, Seoul National University Hospital, Seoul National University College of Medicine, Seoul, Republic of Korea

**Keywords:** Colon cancer, Cancer microenvironment, Prognostic markers

## Abstract

Tumor-infiltrating lymphocytes (TIL) have been suggested as an important prognostic marker in colorectal cancer, but assessment usually requires additional tissue processing and interpretational efforts. The aim of this study is to assess the clinical significance of artificial intelligence (AI)-powered spatial TIL analysis using only a hematoxylin and eosin (H&E)-stained whole-slide image (WSI) for the prediction of prognosis in stage II–III colon cancer treated with surgery and adjuvant therapy. In this retrospective study, we used Lunit SCOPE IO, an AI-powered H&E WSI analyzer, to assess intratumoral TIL (iTIL) and tumor-related stromal TIL (sTIL) densities from WSIs of 289 patients. The patients with confirmed recurrences had significantly lower sTIL densities (mean sTIL density 630.2/mm^2^ in cases with confirmed recurrence vs. 1021.3/mm^2^ in no recurrence, *p* < 0.001). Additionally, significantly higher recurrence rates were observed in patients having sTIL or iTIL in the lower quartile groups. Risk groups defined as high-risk (both iTIL and sTIL in the lowest quartile groups), low-risk (sTIL higher than the median), or intermediate-risk (not high- or low-risk) were predictive of recurrence and were independently associated with clinical outcomes after adjusting for other clinical factors. AI-powered TIL analysis can provide prognostic information in stage II/III colon cancer in a practical manner.

## Introduction

The standard treatment of resectable colon cancer consists of surgery with or without adjuvant therapy, guided by the TNM staging system^1^. Although advances in screening, surgical techniques, and adjuvant therapies led to substantial improvement in outcomes of patients diagnosed with colon cancer, approximately 20–30% of stage II–III colon cancer patients are estimated to relapse^2,3^, and there remains significant variability in clinical outcomes among patients in the same risk categories, emphasizing the need for more precise prognostic biomarkers.

Tumor-infiltrating lymphocytes (TIL) in the tumor microenvironment, which can reflect the host’s immune response to the tumor, have long been recognized as a biomarker that can be related to cancer prognosis^4–6^, and have recently been highlighted as a practical prognostic biomarker for colon cancer. Several recent studies have demonstrated that higher densities of TIL in tumors or their surroundings are associated with better prognoses after standard therapies^7–11^. The most representative example is the “Immunoscore”, which is a scoring system that utilizes CD3+ and CD8+ immune cell densities in the tumor core and the invasive margin, by dedicated software. The assay’s prognostic value has been proven in a large-scale, international cohort of colorectal cancer patients^7,12^. Although other studies evaluating the prognostic value of TILs in colorectal cancer have employed various methodologies concerning the selection of T-cell subsets and the specific spatial regions of the tumor microenvironment to assess the TIL densities, they have converged on similar conclusions regarding the prognostic significance of TILs^11^. While there remains no definitive method for quantification of TILs, evaluating TIL densities in the tumor microenvironment may provide clinicians with additional prognostic information to guide treatment decisions and ultimately improve patient outcomes. However, the manual evaluation of TIL densities can be a laborious and time-consuming process and can also be prone to inter- and intra-observer variations^13,14^. Additionally, it may require additional steps in tissue preparation such as special staining for lymphocytes^15,16^. Even with the more established “Immunoscore”, there is no global consensus, particularly regarding the cut-off values distinguishing the high or low CD3 or CD8 densities.

To overcome such limitations, there has been a growing interest in developing automated methods for TIL evaluation. The continued development and advances in artificial intelligence (AI) technologies, particularly those involving deep learning techniques, present the possibility of automated analysis of intricate visual data sources, such as hematoxylin and eosin (H&E)-stained histopathological images. The convolutional neural network (CNN) is the most representative deep learning model that is being applied to medical image analysis. It can automatically and adaptively learn features from images to accomplish tasks such as classification, detection, and segmentation^17,18^. Early AI-based approaches performed TIL evaluation by classifying TIL density at the tile or patch level, and then constructing maps of TIL scores for analysis at the full WSI level^19^. Concerns on the accuracy and interpretability of such methods for TIL evaluation, especially in terms of capturing the broader context of the entire WSI^20,21^ have led to the development of deep learning methods that classify individual cells^22^. AI-based methodologies are also capable of segmenting areas of interest, such as cancerous regions from medical images including pathology slides^23^. By analyzing each cell and region in the aforementioned manners, explicit and fine-grained measurements of TIL density can be made, improving the accuracy and reliability of AI-based TIL analysis. Such analysis using AI could provide efficiency and improve the reproducibility and thus reliability of TIL as a biomarker.

Lunit SCOPE IO is an AI-powered spatial TIL analyzer, based on CNN models that include both the cell detection and tissue segmentation AI models^24,25^. It was developed and trained using a significant volume of pathology images annotated by board-certified pathologists. The cell detection AI model identifies the location of tumor cells and lymphocytes, while the tissue segmentation AI model determines whether a pixel belongs to a cancer area, cancer stroma, or a non-tumor background region. The system recognizes TILs within spatial segmentation contexts from H&E-stained whole slide images (WSI) and quantifies TILs to calculate TIL densities in two areas of interest: 1) intratumoral TIL (iTIL) density, and 2) tumor-related stromal TIL (sTIL) density. Using the spatial TIL density information, it can also derive immune phenotypes of each WSI, which was defined and shown to be correlated with local immune cytolytic activities in a previous study^26^. The correlation between the TIL assessments and immune phenotyping by Lunit SCOPE IO and clinical outcomes to immune checkpoint inhibitors (ICI) were reported in non-small cell lung cancer and nasopharyngeal carcinoma as well as in a retrospective study of ICI-treated populations with any cancer types, showing that the results can predict clinical outcomes such as survival and response to immunotherapies^26–28^.

In this study, we aimed to evaluate the clinical utility of AI-powered spatial TIL analysis for predicting the prognosis of stage II–III colon cancer patients who underwent curative resection and adjuvant chemotherapy.

## Results

### Patient characteristics and overview of spatial TIL analysis

A total of 289 patients and their WSIs of primary colon cancer tissues were included in this analysis. The clinical characteristics of the included patients are summarized in Table 1. Overall, the median age of the patients was 64 years (interquartile range [IQR] 54–70), and 165 (57.1%) patients were male. 108 (37.4%) patients had stage II and 181 (62.6%) patients had stage III disease. Ninety patients (31.1%) had T4 or N2 disease, and 131 (45.3%) and 121 (41.9%) patients exhibited lymphovascular or perineural invasion, respectively. The median follow-up duration of the included patients was 8.0 years (IQR 5.8–9.8 years), and 91.3% (232/254) of the patients without any events of interest (tumor recurrence or death) were followed for at least 5.0 years. During the follow-up period, 28 (9.7%) clinical recurrences and 23 (8.0%) death events were observed, including 7 deaths not related to colon cancer.

**Table 1:** Patient characteristics.

Characteristics	All patients
	*N* = 289
Age, median (interquartile range)	64 (54–70)
Sex	
Male	165 (57.1%)
Female	124 (42.9%)
Primary site of disease^a^	
Right colon	90 (31.1%)
Left colon	199 (68.9%)
Stage	
II	108 (37.4%)
Stage II with high-risk features^b^	70 (24.2%)
III	181 (62.6%)
T stage	
T1–3	236 (81.7%)
T4	53 (18.3%)
N stage	
N0–1	238 (82.4%)
N2	51 (17.6%)
T4 or N2 disease	90 (31.1%)
Histology	
Adenocarcinoma, not otherwise specified	282 (97.6%)
W/D	17 (5.9%)
M/D	234 (81.0%)
P/D	31 (10.7%)
Mucinous adenocarcinoma	7 (2.4%)
Lymphovascular invasion	
No	158 (54.7%)
Yes	131 (45.3.%)
Perineural invasion	
No	168 (58.1%)
Yes	121 (41.9%)
Microsatellite instability	
MSS/MSI-L	262 (90.7%)
MSI-H	20 (6.9%)
NA	7 (2.4%)
Adjuvant chemotherapy regimens	289 (100.0%)
FL/Capecitabine/Tegafur-uracil	126 (43.6%)
FOLFOX/XELOX	163 (56.4%)

*W/D* well-differentiated, *M/D*: moderately-differentiated, *P/D* poorly differentiated, *MSS* microsatellite stable, *MSI-L* microsatellite instability-low, *MSI-H* microsatellite instability-high, *FL* 5-fluorouracil + leucovorin, *FOLFOX* 5-fluorouracil + leucovorin + oxaliplatin, *XELOX* capecitabine + oxaliplatin.

^a^Right-sided colon cancer: cecum, ascending, hepatic flexure, or transverse colon; left-sided colon cancer: splenic flexure, descending, sigmoid, or rectosigmoid colon.

^b^High-risk features in stage II patients were defined as having one or more of the following: T4 disease, poorly differentiated histology, lymphovascular/perineural invasion, bowel obstruction or perforation, fewer than 12 lymph nodes harvested or high initial plasma carcinoembryonic antigen (CEA) level (>5 ng/mL).

In all patients, the TILs within the tumor microenvironment were predominantly found to be localized in the stroma, with the median sTIL density of 878.0/mm^2^ (IQR 554.9–1209.6/mm^2^) and the median iTIL density of 44.4/mm^2^ (IQR 28.4–71.8/mm^2^) (Supplementary Table 1). The sTIL densities showed a strong positive correlation with the average of the TIL scores estimated by two pathologists in accordance with the International TILs Working Group (ITWG) guideline (Spearman’s *r* = 0.820, *p* < 0.001, Supplementary Fig. 1). The densities of iTIL and sTIL showed a modest positive correlation as continuous variables (Spearman’s *r* = 0.464, *p* < 0.001). Distribution of the mean iTIL and sTIL densities according to clinicopathologic risk factors are summarized in Table 2. The iTIL and sTIL densities were significantly lower in patients with stage III disease compared to stage II (iTIL 58.3/mm^2^ in stage III vs. 79.5/mm^2^ in stage II, *p* = 0.046; sTIL 926.4/mm^2^ in stage III vs. 1079.0/mm^2^ in stage II, *p* = 0.049). Additionally, the patients having T4 or N2 disease or perineural invasion showed significantly lower sTIL densities (sTIL 844.9/mm^2^ in T4 or N2 disease vs. 1046.1/mm^2^ in others, *p* = 0.007; sTIL 849.2/mm^2^ with perineural invasion vs. 1080.1/mm^2^ without perineural invasion, *p* = 0.001). The tumors with high microsatellite instability (MSI-H) exhibited significantly higher infiltrations of TIL intratumorally (iTIL 161.5/mm^2^ in MSI-H vs. 58.9/mm^2^ in MSI-L/MSS, *p* = 0.024) but not in their stroma, and similar iTIL differences were also observed in right-sided tumors vs. left (iTIL 85.4/mm^2^ in right-sided tumors vs. 57.6/mm^2^ in left-sided tumors, *p* = 0.030), and poorly differentiated (P/D) tumors vs. others (iTIL 149.4/mm^2^ in P/D tumors vs. 56.3/mm^2^ in others, *p* = 0.004).

**Table 2:** Distribution of iTIL and sTIL densities by clinicopathologic variables.

	iTIL (/mm^2^) mean (SD)	*p*-value	sTIL (/mm^2^) mean (SD)	*p*-value
Age				
<64	68.1 (72.6)	0.695	1041.9 (696.6)	0.108
≥64	64.4 (86.1)		925.3 (518.8)	
Stage				
II	79.5 (96.8)	0.046	1079.0 (662.4)	0.049
III	58.3 (66.3)		926.4 (580.2)	
T stage				
T1–3	64.7 (75.8)	0.553	1018.2 (608.5)	0.050
T4	73.0 (94.9)		828.6 (628.7)	
N stage				
N0–1	66.4 (78.4)	0.943	1016.8 (648.8)	0.008
N2	65.5 (85.4)		827.8 (396.6)	
T4 or N2 disease				
No	65.4 (77.9)	0.789	1046.1 (632.8)	0.007
Yes	68.2 (83.4)		844.9 (554.1)	
Tumor location				
Right	85.4 (113.7)	0.030	1022.5 (590.7)	0.459
Left	57.6 (56.2)		965.7 (627.1)	
MSI status				
MSI-H	161.5 (186.3)	0.024	1109.9 (582.3)	0.312
MSS/MSI-L	58.9 (60.5)		969.4 (620.1)	
Lymphovascular invasion				
No	70.6 (89.5)	0.290	1023.6 (636.7)	0.220
Yes	60.9 (65.5)		934.9 (587.7)	
Perineural invasion				
No	72.2 (88.8)	0.114	1080.1 (669.1)	0.001
Yes	58.0 (63.9)		849.2 (505.0)	
Differentiation^a^ (all patients)				
High-grade	149.4 (163.2)	0.004	1168.0 (790.4)	0.168
Low-grade	56.3 (55.2)		961.3 (589.1)	
Differentiation^a^ (MSS/MSI-L)				
High-grade	111.8 (123.8)	0.054	1060.7 (904.2)	0.494
Low-grade	54.6 (50.0)		961.8 (592.6)	

*SD* standard deviation, *iTIL* intratumoral tumor-infiltrating lymphocyte, *sTIL* stromal tumor-infiltrating lymphocyte, *MSI* microsatellite instability, *MSS* microsatellite stable; *MSI-L* microsatellite instability-low, *MSI-H* microsatellite instability-high.

^a^High-grade tumor differentiation includes poorly differentiated or undifferentiated tumors; low-grade includes well- or moderately-differentiated tumors.

### Spatial TIL analysis in association with clinical outcomes

**Fig. 1:**
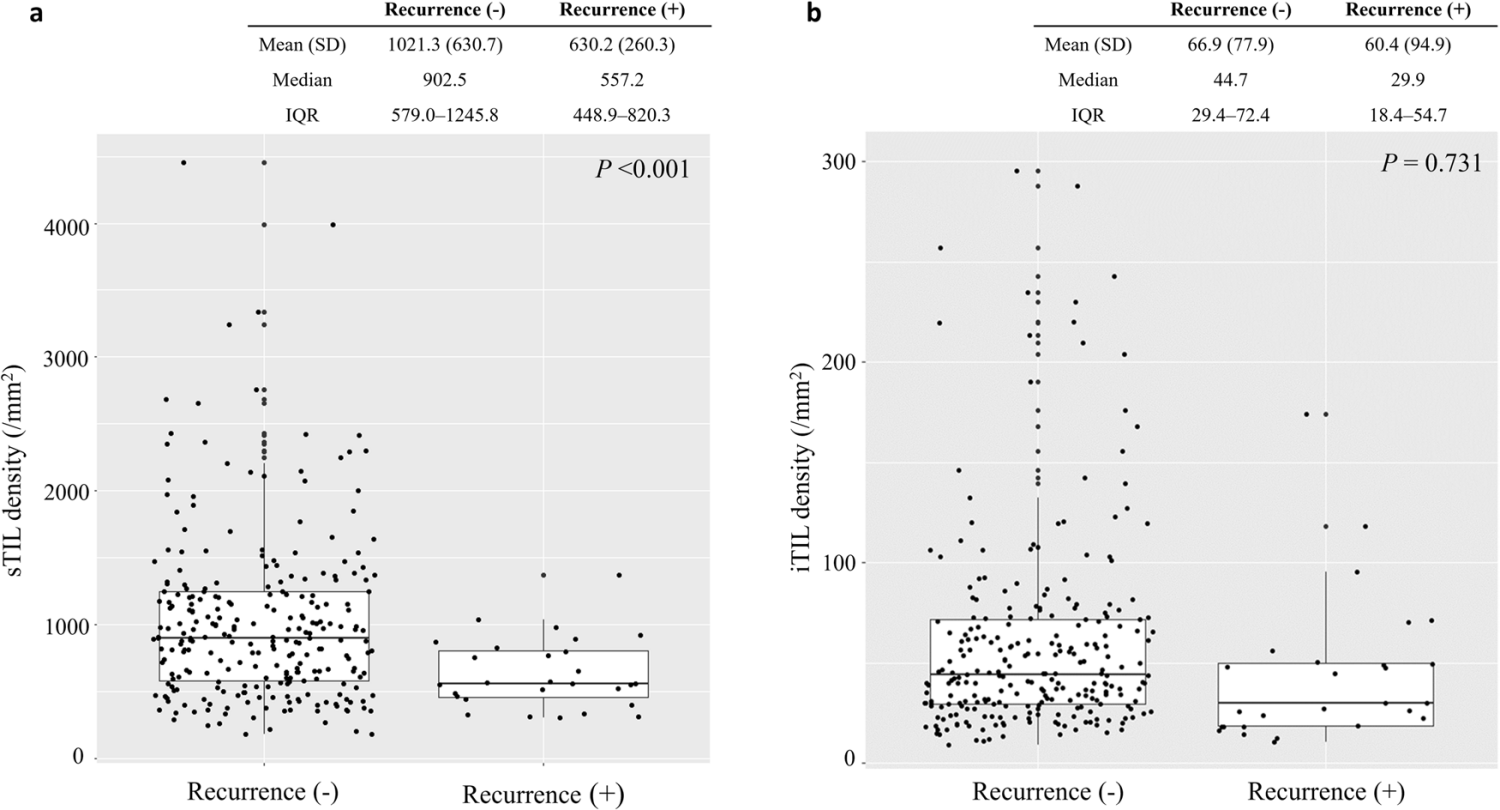
The distribution of tumor-infiltrating lymphocyte densities according to recurrence events. The distribution of (**a**) tumor-related stromal (sTIL) and (**b**) intratumoral (iTIL) tumor-infiltrating lymphocyte densities according to recurrence events. In the plot, the upper and lower boundaries of the box represent the upper and lower quartiles, while the line inside the box represents the median of the data (Recurrence (+), cases with confirmed recurrences; Recurrence (-), cases with no recurrence events during the follow-up period; SD, standard deviation; IQR, interquartile range).

When the spatial TIL densities were analyzed in relation to clinical outcomes, the sTIL densities were significantly lower in the 28 patients with confirmed recurrences (mean sTIL 630.2/mm^2^ in cases with confirmed recurrences vs. 1021.3/mm^2^ in no recurrence, *p* < 0.001, Fig. 1a). However, the difference in the mean iTIL densities was not prominent by clinical recurrences (mean iTIL 60.4/mm^2^ in cases with confirmed recurrences vs. 66.9/mm^2^ in no recurrence, *p* = 0.731, Fig. 1b).

**Fig. 2:**
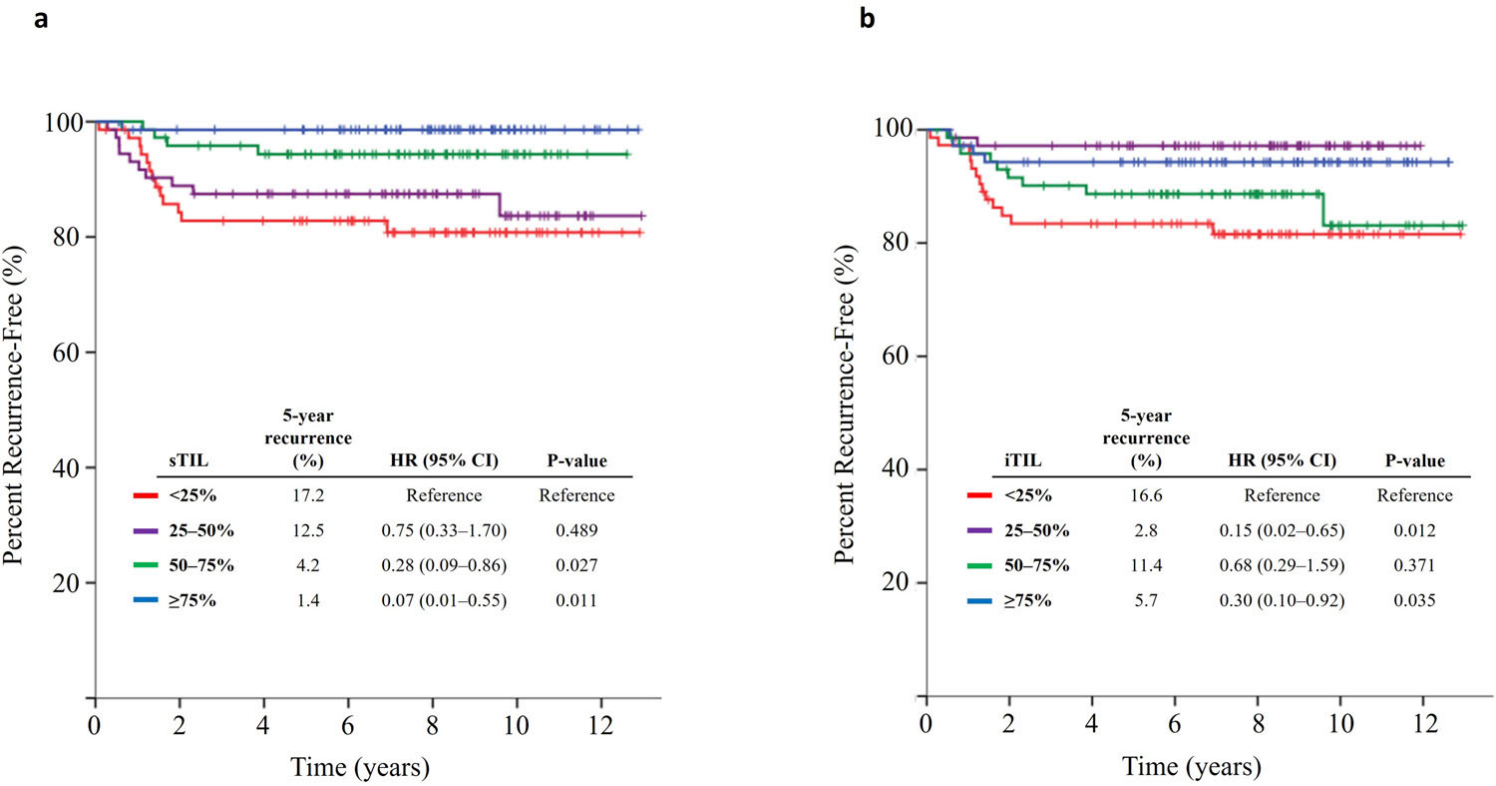
Kaplan Meier curves of time to recurrence (TTR) according to tumor-infiltrating lymphocyte densities. Kaplan Meier curve of TTR according to (**a**) tumor-related stromal tumor-infiltrating lymphocyte (sTIL) densities and (**b**) intratumoral tumor-infiltrating lymphocyte (iTIL) densities. In Fig. 2b, when patient groups with iTIL densities ≥25% were combined together, the recurrence rate at 5 years was 6.7%. The hazard ratio (HR) of recurrence in the combined group (iTIL ≥25% vs. <25%) was 0.37 (95% confidence interval [CI] 0.18–0.78; *p* = 0.009).

By dividing the patients by sTIL densities into four groups using quartile cutoffs, the recurrence rate at 5 years was observed to be the lowest in the highest quartile group (1.4%) and increased with decreasing sTIL densities (4.2%, 12.5% and 17.2% in the 50–75%, 25–50%, and <25% groups, respectively), with the unadjusted hazard ratio (HR) of time to recurrence (TTR) for the highest vs. the lowest quartile groups of 0.07 (95% CI 0.01–0.55, *p* = 0.011, Fig. 2a). Furthermore, a similar pattern was observed in disease-free survival (DFS), with the most substantial differences between groups observed at the median value of sTIL densities (5-year DFS rate 94.4% in sTIL ≥50% vs. 83.3% in sTIL <50% [log-rank *p* = 0.001], Supplementary Table 2).

Analysis with iTIL densities showed that the recurrence rate at 5 years was significantly higher in the iTIL <25% group, with a recurrence rate of 16.6% (vs. 6.7% in the rest [iTIL ≥25%], with unadjusted HR of 0.37 in iTIL ≥25% vs. <25%, 95% CI 0.18–0.78; *p* = 0.009). Unlike the sTIL quartile groups, the recurrence risk did not sequentially increase with the reduction in the quartile value of iTIL densities in the iTIL ≥25% groups (Fig. 2b). Combining the iTIL to sTIL quantification did not add value to recurrence prediction in sTIL ≥25% groups, but in the lowest sTIL group ( < 25%), the recurrence was significantly higher if iTIL was also <25% (5-year recurrence rate of 26.1% [in sTIL <25% and iTIL <25%] vs. 10.3% [in sTIL < 25% and iTIL ≥ 25%]; *p* = 0.044).

### Combined iTIL/sTIL risk groups for prediction of prognoses

**Fig. 3:**
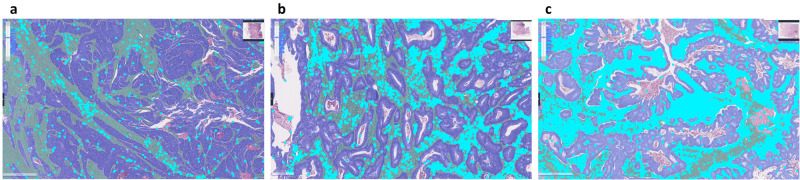
Representative images of Lunit SCOPE IO-inferenced hematoxylin and eosin-stained whole slide images. The representative image of a Lunit SCOPE IO-inferenced whole slide image in, (**a**) a high-risk case, (**b**) an intermediate-risk case, and (**c**) a low-risk case (blue: cancer area, green: cancer stroma, cyan dots: tumor-infiltrating lymphocytes. Unmarked areas in the whole slide images refer to background area not directly related to either cancer area or the cancer related stroma).

**Fig. 4:**
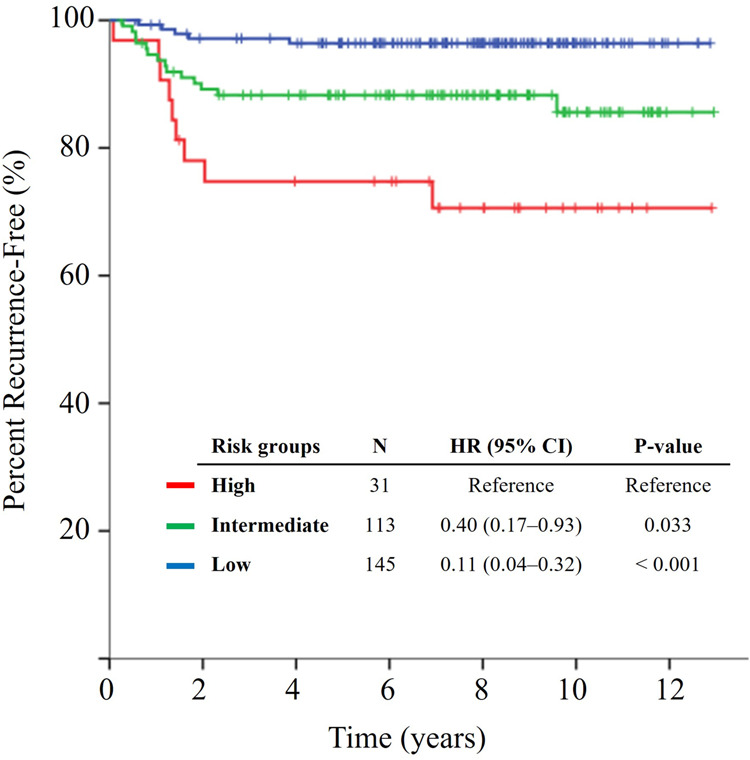
Kaplan Meier curves of time to recurrence (TTR) according to combined iTIL/sTIL Risk Groups. The Kaplan Meier curves of TTR according to the combined intratumoral (iTIL)/tumor-related stromal (sTIL) tumor-infiltrating lymphocyte risk groups (HR, hazard ratio; CI, confidence interval).

Based on the analysis of iTIL and sTIL results in predicting recurrences and survival outcomes, we defined three recurrence risk groups using the combined sTIL and iTIL values: high-risk (both iTIL <25% and sTIL <25%), low-risk (sTIL ≥50% with any iTIL), and intermediate-risk (not meeting the criteria for high or low; Fig. 3). Using these categorization cutoffs, 31 (10.7%), 113 (39.1%), and 145 (50.2%) patients were grouped into high-risk, intermediate-risk, and low-risk, respectively. The combined three risk group categorization significantly stratified patients for TTR (*p* < 0.001, Fig. 4) and DFS (*p* < 0.001, Supplementary Fig. 2). The three-risk group categorization remained effective in stratifying patients in subgroups of right-sided and left-sided tumors, or in subgroups of stage II and stage III (Supplementary Table 3). In the multivariable analysis for TTR or DFS adjusting for the age, sex, T, and N stages, tumor differentiation, lymphovascular/perineural invasion, and tumor sidedness, the combined TIL risk groups were shown to be significantly and independently associated with the clinical outcomes (Table 3).

**Table 3:** Multivariable analysis^a^ of time to recurrence and disease-free survival according to TIL risk groups, adjusted for other clinicopathologic variables.

	Time to recurrence		Disease-free survival	
	HR (95% CI)	*p*-value	HR (95% CI)	*p*-value
TIL risk categories				
High-risk	1	0.010	1	0.018
Intermediate-risk	0.41 (0.18–0.96)	0.040	0.49 (0.22–1.09)	0.081
Low-risk	0.18 (0.06–0.56)	0.003	0.25 (0.10–0.65)	0.004
Age, per year increase	1.02 (0.98–1.06)	0.285	1.03 (1.00–1.07)	0.070
Sex				
Male	1	0.406	1	0.184
Female	1.40 (0.63–3.13)		1.64 (0.79–3.41)	
T stage				
T4	1	< 0.001	1	< 0.001
T1–3	0.16 (0.07–0.35)		0.18 (0.09–0.37)	
N stage				
N2	1	0.007	1	0.004
N0–1	0.34 (0.16–0.74)		0.35 (0.17–0.71)	
Tumor differentiation^b^				
High-grade	1	0.172	1	0.212
Low-grade	0.50 (0.19–1.35)		0.56 (0.22–1.40)	
Lymphovascular/perineural invasion				
Yes	1	0.067	1	0.020
No	0.36 (0.12–1.08)		0.31 (0.12–0.83)	
Tumor sidedness				
Right	1	0.872	1	0.283
Left	0.93 (0.41–2.14)		0.68 (0.34–1.37)	

*HR* hazard ratio, *CI* confidence interval, *TIL* tumor-infiltrating lymphocytes.

^a^Microsatellite instability status was not included in the multivariate analysis as there were no recurrence events observed in the 20 patients having microsatellite instability-high tumors.

^b^High-grade tumor differentiation includes poorly differentiated or undifferentiated tumors; low-grade includes well- or moderately-differentiated tumors.

## Discussion

In this study, we have investigated the potential of AI-powered spatial TIL analysis for predicting prognosis in patients with colon cancer treated with surgery and adjuvant therapy. In the context of stage II–III colon cancer, the infiltration of TILs in the tumor microenvironment was predominantly in the stroma surrounding the tumor and the densities of sTIL demonstrated a significant association with patient prognosis. The density of iTIL was found to have a lower mean density and exhibited less variance compared to sTIL, but the lowest quartile of iTIL densities was also found to be related to higher risk of recurrence.

In recent years, there has been a growing interest in evaluating TILs as a prognostic factor in solid tumors. In colorectal cancer, numerous studies have been conducted to explore the prognostic significance of TILs, encompassing both general and marker-selected subsets, and in various locations within the tumor microenvironment, such as the tumor center, invasive margin, or the surrounding stroma^7,10,11^. The large-scale validation of ‘Immunoscore’, which quantifies CD3+ and CD8+ lymphocytes in the tumor center and invasive margin, showed that Immunoscore could predict the risk of recurrence with higher risk contribution than other clinical parameters including the TNM classification system^7^. The ITWG suggested a standardized approach for evaluating the degree of TIL infiltration in breast cancer, by assessing stromal TILs as a percentage of stromal area occupied by the TILs^29,30^, and the evaluation of TILs with the same scoring method proved to be prognostic in colorectal cancer as well^31^. Despite the existence of various evaluation methods for assessing TILs, the studies of TILs in relation to clinical outcomes consistently indicate that TILs may serve as an independent prognostic biomarker in colon cancer and highlights the necessity for developing efficient and reliable assessment techniques.

Recent advances in deep learning technologies have facilitated the development of AI-based methodologies that can extract features from medical images. Deep learning models used for medical images, especially pathology images, need to be trained on relatively limited data, due to the inherent challenge of obtaining reliable ground-truth annotation by experts. Our model used one of the well-established CNN architectures. While alternative feature extractors could be utilized, newer structures, such as those based on Vision Transformer architectures, may not offer benefits over more traditional designs when there are constraints on the number of inputs.

A key advantage of developing AI-based methods for TIL evaluation is that it can provide consistent and reproducible outcomes, in addition to streamlining the labor-intensive work process. The consistent evaluation by AI-based methods can be particularly beneficial in situations such as the evaluation of iTILs in our dataset, where the densities are low and within a restricted range. In such cases, even small differences in TIL counting caused by subjective variations in human observation can lead to a considerable disparity in evaluating the case to have high or low TIL densities. This underscores the value of an AI-based method that can provide a consistent evaluation process.

Our methodology of assessing the degree of TIL infiltrations by using only the H&E-stained images has advantage of not requiring any additional procedures such as immunohistochemistry (IHC), thus simplifying the methodology and offering wide applicability. Furthermore, the straightforward nature of the process reduces the likelihood of introducing artifacts that may be caused by additional experimental steps^32,33^. However, this approach does not consider the subtypes of lymphocytes^4,11,34^. Instead, we incorporated spatial information between the tumor, stroma, and TILs in our analysis. Our findings demonstrate that while stromal TILs play a significant role in the prognostic prediction of colon cancer, intratumoral TILs can also aid in identifying patients with particularly poor prognoses. Although positive correlations with clinical outcomes were observed in this study, future research may be necessary to improve the predictive accuracy through the incorporation of additional features, such as more detailed spatial boundary delineation by utilizing the distance from the tumor center or the invasive border. In addition, there exists a need to validate the prognostic value of our spatial TIL-based risk groups using fixed cutoff values with an expanded, multicentric dataset in order to have the model applicable to the clinical setting. Nevertheless, our results hold significance in that they demonstrate, although in a pilot stage, that predicting clinical outcomes through the utilization of an AI-powered model could aid in standardizing the evaluation process and streamlining the workload.

The 5-year recurrence rate of 9.7% observed in the patients included in this study is notably lower than the widely reported recurrence rate of 20–30% for this patient population. All patients included in this study were treated at Seoul National University Bundang Hospital (SNUBH). SNUBH is the first fully digitalized paperless hospital in Korea from its beginning in 2003, and all patients’ clinical and radiologic data at SNUBH have been electronically recorded and maintained in the electronic medical record system. In addition, clinical data of colorectal cancer patients who underwent surgery have been collected and maintained by constructing a prospective database in the department of surgery at SNUBH^35^. Although the data analysis of this study was conducted retrospectively, patients’ data had already been collected prospectively based on the above databases, and cancer recurrence was reviewed once again for this study. All patients in our analysis underwent surgery and received adjuvant chemotherapy as appropriate according to clinical practice guidelines at the time. Most (89.6%) patients had completed their adjuvant therapy as planned. Among all included patients, thirty-eight patients (13.1%) had low-risk stage II disease. In case of low-risk stage II colon cancer, either adjuvant chemotherapy or observation without chemotherapy can be considered because of modest survival benefit^1^; in these patients with low-risk stage II disease, after the shared discussion of the actual expected benefit of adjuvant chemotherapy, fluoropyrimidine monotherapy was used in all cases who wanted adjuvant treatment in our patient cohort. Apart from the pathologic stage and the treatment factor, patient selection was based solely on the availability of slides for the WSI analysis and not on any other criteria, thereby reflecting the comprehensive patient population during the predetermined treatment period (2009–2012). Moreover, we were able to gather the follow-up information for enough time in most of the included patients. Probably, the fact that all patients included in this study received adjuvant chemotherapy and showed high compliance with adjuvant treatment may partially explain the good treatment results compared to other reports. Therefore, we believe that the reported outcomes on cancer recurrence and survival in this study reflected the actual reality at SNUBH. Since the analysis in this study is based on single-institution patient data, we emphasize once again that validation is required in the future.

In conclusion, AI-powered TIL analysis has the potential to serve as a robust and practical tool to provide prognostic information in stage II–III colon cancer. Further validation in a larger number of cases is necessary to establish the full extent of its applicability.

## Methods

### Study patients and data sets

First, patients with pathologic stage II or III colon cancer who were treated with curative surgery between Jan 2009 and Dec 2012 at SNUBH were selected in this retrospective study. Among these, patients who received adjuvant chemotherapy and had available H&E tumor slides were finally included in this study (*N* = 289). All patients received fluoropyrimidine-based adjuvant therapy (with or without oxaliplatin). WSIs of H&E-stained primary tumor tissues prepared from formalin-fixed paraffin-embedded samples obtained at the time of surgery were scanned at a 40x magnification using Aperio AT2 (Leica Microsystems Inc, Buffalo Grove, IL, USA) for the AI-based spatial TIL analysis. A single H&E-stained WSI of a representative primary tumor tissue block was selected and scanned for the TIL analysis of each patient.

The study was conducted in accordance with the Declaration of Helsinki for biomedical research. The Institutional Review Board of SNUBH approved this study and obtaining informed consent from individual patients was waived considering the retrospective nature of this study (B-2110-716-302).

### Spatial TIL analysis by AI-powered WSI analyzer

Lunit SCOPE IO (Lunit Inc., Seoul, Republic of Korea) is a deep learning-based TIL analyzer, comprised of two complementary but separate deep learning models each developed for cell detection and for tissue segmentation, as previously described (Supplementary Fig. 3)^26,27,36^. The deep learning models are based on the DeepLabV3+ convolutional neural network architecture, with a ResNet-34 backbone network^37,38^. The models were developed and trained with patches extracted from WSIs of 25 tumor types including colon cancer, annotated and segmented by board-certified pathologists and were updated from a previous version^26^ using 13.5 × 10^9^ µm^2^ tissue regions and 6.2 × 10^5^ TILs on 17,292 H&E-stained WSIs from 17 tumor types also including colon cancer. The performance of the model, assessed using the tuning dataset prior to and independently of its application to the dataset of this study, yielded the intersection over union (IoU) of 0.82 and 0.67 for the model’s capacity of segmentation of cancer area and cancer stroma respectively, and mF1 score of 0.71 for the detection of TILs or tumor cells. To ascertain the performance of Lunit SCOPE IO on segmenting and detecting TIL in colon cancer, the performance was separately validated using samples included in The Cancer Genome Atlas (TCGA) colon adenocarcinoma (COAD) dataset. An experienced pathologist (H.J.O.) annotated 106 tumor-containing test grids randomly selected from the WSI in the TCGA COAD dataset for the ground-truth of tissue segmentation and TIL identification. The segmentation and TIL identification results of Lunit SCOPE IO were compared to the pathologist’s annotation to evaluate the performance of the model, yielding the IoU of 0.84 for segmentation of cancer area, 0.85 for segmentation of cancer stroma, and mF1 score of 0.71 for TIL detection.

For this study, Lunit SCOPE IO quantified TIL and combined the spatial segmentation data with the location data of lymphocytes on a WSI. iTIL and sTIL densities, defined as the number of TILs per 1 mm^2^ of cancer area or the cancer stroma were obtained for analysis in association with clinical outcomes. To compare the TIL densities determined by Lunit SCOPE IO to the results using a pre-existing model, two pathologists (H.J.O. and C.K.) independently scored the TIL densities of the same dataset included in this study using the standardized approach suggested by the ITWG^29,30^. The ITWG TIL scores examined by the two pathologists were compared to the sTIL densities calculated by Lunit SCOPE IO, as the ITWG method specifies to evaluate only the TILs within the stromal compartment.

### Statistical analysis

Differences in means for continuous variables between the two groups were compared using the Wilcoxon rank-sum test. The categorical variables between the two groups were compared using the Chi-square test and Fisher’s exact test was applied if the expected frequencies in >20% of the cells were below 5. The correlation between two continuous variables was assessed using Spearman’s rank correlation coefficient. TTR was defined as the time from surgery to confirmation of recurrence (distant or locoregional), and DFS was defined as the time from surgery to confirmation of recurrence or death from any cause. Patients alive without an event of interest were censored at the date of the last follow-up visit. The univariate comparisons of TTR or DFS were performed using the log-rank tests, and the multivariate comparisons were performed using the Cox proportional hazard model. Two-sided p-values were reported and p-values of less than 0.05 were considered statistically significant. The statistical analysis was performed using R version 4.2.2 (www.r-project.org).

### Reporting summary

Further information on research design is available in the Nature Research Reporting Summary linked to this article.

### Supplementary information

Supplementary Tables 1-3, Supplemnetary Figures 1-3

REPORTING SUMMARY

## Data Availability

The AI-powered analysis results of spatial TIL density data generated during this study are included in this article and the supplementary information files. The other data will be made available upon reasonable request to the corresponding author.

## Code availability

All scripts implemented in R 4.2.2 are available for noncommercial academic purposes upon reasonable written request.

## References

[1] National Comprehensive Cancer Network. *Colon Cancer (Version 3, 2022)*. https://www.nccn.org/professionals/physician_gls/pdf/colon.pdf (2022).

[2] Cho JR (2022). Effectiveness of oral fluoropyrimidine monotherapy as adjuvant chemotherapy for high-risk stage II colon cancer. Ann. Surg. Treat. Res..

[3] Miller KD (2022). Cancer treatment and survivorship statistics, 2022. CA Cancer J. Clin..

[4] Galon J (2006). Type, density, and location of immune cells within human colorectal tumors predict clinical outcome. Science.

[5] Naito Y (1998). CD8+ T cells infiltrated within cancer cell nests as a prognostic factor in human colorectal cancer. Cancer Res..

[6] Pages F (2005). Effector memory T cells, early metastasis, and survival in colorectal cancer. N. Engl. J. Med..

[7] Pages F (2018). International validation of the consensus Immunoscore for the classification of colon cancer: a prognostic and accuracy study. Lancet.

[8] Mlecnik B (2020). Multicenter international society for immunotherapy of cancer study of the consensus immunoscore for the prediction of survival and response to chemotherapy in stage III colon cancer. J. Clin. Oncol..

[9] Rozek, L. S. et al. Tumor-infiltrating lymphocytes, Crohn’s-like lymphoid reaction, and survival from colorectal cancer. *J. Natl Cancer Inst.*10.1093/jnci/djw027 (2016). 10.1093/jnci/djw027 PMC5017930 27172903

[10] Lee H (2020). Analysis of tumor microenvironmental features to refine prognosis by T, N risk group in patients with stage III colon cancer (NCCTG N0147) (Alliance). Ann. Oncol..

[11] Idos GE (2020). The prognostic implications of tumor infiltrating lymphocytes in colorectal cancer: a systematic review and meta-analysis. Sci. Rep..

[12] Pages F (2020). Prognostic and predictive value of the Immunoscore in stage III colon cancer patients treated with oxaliplatin in the prospective IDEA France PRODIGE-GERCOR cohort study. Ann. Oncol..

[13] Khoury T, Peng X, Yan L, Wang D, Nagrale V (2018). Tumor-infiltrating lymphocytes in breast cancer: evaluating interobserver variability, heterogeneity, and fidelity of scoring core biopsies. Am. J. Clin. Pathol..

[14] Swisher SK (2016). Interobserver agreement between pathologists assessing tumor-infiltrating lymphocytes (TILs) in breast cancer using methodology proposed by the International TILs Working Group. Ann. Surg. Oncol..

[15] Chaput N (2009). Identification of CD8+CD25+Foxp3+ suppressive T cells in colorectal cancer tissue. Gut.

[16] Ali HR (2014). Association between CD8+ T-cell infiltration and breast cancer survival in 12,439 patients. Ann. Oncol..

[17] Yu H, Yang LT, Zhang Q, Armstrong D, Deen MJ (2021). Convolutional neural networks for medical image analysis: State-of-the-art, comparisons, improvement and perspectives. Neurocomputing.

[18] Sarvamangala DR, Kulkarni RV (2022). Convolutional neural networks in medical image understanding: a survey. Evol. Intell..

[19] Saltz J (2018). Spatial organization and molecular correlation of tumor-infiltrating lymphocytes using deep learning on pathology images. Cell Rep..

[20] Klauschen F (2018). Scoring of tumor-infiltrating lymphocytes: from visual estimation to machine learning. Semin. Cancer Biol..

[21] Amgad M (2020). Report on computational assessment of Tumor Infiltrating Lymphocytes from the International Immuno-Oncology Biomarker Working Group. NPJ Breast Cancer.

[22] Wang S (2019). ConvPath: a software tool for lung adenocarcinoma digital pathological image analysis aided by a convolutional neural network. EBioMedicine.

[23] Xu J, Luo X, Wang G, Gilmore H, Madabhushi A (2016). A Deep Convolutional Neural Network for segmenting and classifying epithelial and stromal regions in histopathological images. Neurocomputing.

[24] Pantanowitz L (2020). Accuracy and efficiency of an artificial intelligence tool when counting breast mitoses. Diagn. Pathol..

[25] Paeng, K., Hwang, S., Park, S. & Kim, M. A unified framework for tumor proliferation score prediction in breast histopathology. In: Cardoso, M, et al. *Deep Learning in Medical Image Analysis and Multimodal Learning for Clinical Decision Support.* Lecture Notes in Computer Science (Springer, Cham). **10553**, 231–239 (2017).

[26] Park S (2022). Artificial intelligence-powered spatial analysis of tumor-infiltrating lymphocytes as complementary biomarker for immune checkpoint inhibition in non-small-cell lung cancer. J. Clin. Oncol..

[27] Jung HA (2022). A phase II study of nivolumab plus gemcitabine in patients with recurrent or metastatic nasopharyngeal carcinoma (KCSG HN17-11). Clin. Cancer Res..

[28] Shen J (2022). The inflamed immune phenotype (IIP): a clinically actionable artificial intelligence (AI)-based biomarker predictive of immune checkpoint inhibitor (ICI) outcomes across >16 primary tumor types. J. Clin. Oncol..

[29] Hendry S (2017). Assessing tumor-infiltrating lymphocytes in solid tumors: a practical review for pathologists and proposal for a standardized method from the International Immunooncology Biomarkers Working Group: Part 1: assessing the host immune response, TILs in invasive breast carcinoma and ductal carcinoma in situ, metastatic tumor deposits and areas for further research. Adv. Anat. Pathol..

[30] Hendry S (2017). Assessing tumor-infiltrating lymphocytes in solid tumors: a practical review for pathologists and proposal for a standardized method from the international immuno-oncology biomarkers working group: Part 2: TILs in melanoma, gastrointestinal tract carcinomas, non-small cell lung carcinoma and mesothelioma, endometrial and ovarian carcinomas, squamous cell carcinoma of the head and neck, genitourinary carcinomas, and primary brain tumors. Adv. Anat. Pathol..

[31] Fuchs TL (2020). Assessment of tumor-infiltrating lymphocytes using International TILs Working Group (ITWG) system is a strong predictor of overall survival in colorectal carcinoma: a study of 1034 patients. Am. J. Surg. Pathol..

[32] Diem K (2015). Image analysis for accurately counting CD4+ and CD8+ T cells in human tissue. J. Virol. Methods.

[33] Wilson, C. M. et al. Challenges and opportunities in the statistical analysis of multiplex immunofluorescence data. *Cancers*10.3390/cancers13123031 (2021). 10.3390/cancers13123031 PMC8233801 34204319

[34] Chen Y (2016). A novel immune marker model predicts oncological outcomes of patients with colorectal cancer. Ann. Surg. Oncol..

[35] Choi S (2011). Different characteristics and prognostic impact of deep-vein thrombosis / pulmonary embolism and intraabdominal venous thrombosis in colorectal cancer patients. Thromb. Haemost..

[36] Cho, H. G. et al. Artificial intelligence-powered whole-slide image analyzer reveals a distinctive distribution of tumor-infiltrating lymphocytes in neuroendocrine neoplasms. *Diagnostics*10.3390/diagnostics12102340 (2022). 10.3390/diagnostics12102340 PMC9600129 36292028

[37] Chen, L.-C., Papandreou, G., Shroff, F. & Adam, H. Rethinking atrous convolution for semantic image segmentation. *arXiv*10.48550/arXiv.1706.05587 (2017)

[38] He K., Zhang, X., Ren, S. & Sun, J. Deep residual learning for image recognition. *Proceedings of the IEEE Conference on Computer Vision and Pattern Recognition (CVPR)*. 770-778 (2016).

