# Supplementary material for: Artificial intelligence-powered spatial analysis of tumor-infiltrating lymphocytes for prediction of prognosis in resected colon cancer
Source: NPJ Precis Oncol. 2023 Nov 20;7:124. doi: 10.1038/s41698-023-00470-0 (PMC10662481; doi:10.1038/s41698-023-00470-0)

Supplementary Table 1. Densities of tumor-infiltrating lymphocytes on intratumoral area (iTIL) and tumor-related stromal area (sTIL) in all included patients

case ID	Cancer area (pixel)	Stroma area (pixel)	Number of lymphocytes on cancer area	Number of lymphocytes on stroma area	iTIL densty (/mm2)	sTIL density (/mm2)
C1	4791396	8837618	2828	84229	60.44	975.95
C2	4424289	5359776	1160	68879	26.85	1315.95
C3	4626379	2572014	863	12351	19.10	491.73
C4	9674116	13860540	2642	78910	27.97	582.98
C5	3547717	4116345	577	31661	16.65	787.61
C6	6417991	8726526	2632	38032	41.99	446.28
C7	3877130	8571948	2658	77348	70.20	923.99
C8	5147321	6028860	25528	60961	507.85	1035.42
C9	4071608	8481365	2007	72282	50.48	872.70
C10	3601515	6425211	1051	36042	29.88	574.41
C11	4116726	8694342	4753	116361	118.23	1370.47
C12	2141006	4352878	564	14053	26.97	330.59
C13	5631751	9653083	3917	52508	71.22	557.01
C14	3525271	10620011	1027	33884	29.83	326.72
C15	6026148	5178460	1061	37968	18.03	750.79
C16	3561756	7395946	898	40585	25.82	561.92
C17	1836645	4770266	884	37205	49.29	798.65
C18	2693596	13252752	4578	126763	174.04	979.46
C19	16074610	13824577	2930	42425	18.66	314.25
C20	7676627	6965553	1772	44632	23.64	656.13
C21	3691279	12988197	1755	56406	48.69	444.71
C22	5868128	12890051	5457	70159	95.23	557.35
C23	3092295	6664750	507	23750	16.79	364.90
C24	2231916	6188572	954	29706	43.77	491.53
C25	3564600	3601977	1895	77507	54.44	2203.43
C26	5576706	7728026	3562	207826	65.41	2753.79
C27	4504973	5378575	2409	44848	54.76	853.84
C28	4697550	7051217	1575	137699	34.33	1999.71
C29	1388375	3760970	691	19393	50.96	528.01
C30	6744844	4075890	821	30635	12.46	769.65
C31	5428781	1383364	2977	7010	56.15	518.90
C32	5015279	10712704	789	50621	16.11	483.87
C33	4769129	5372663	850	21079	18.25	401.75
C34	5351575	6114726	2500	49418	47.84	827.58
C35	5340188	7678325	741	23554	14.21	314.12
C36	3875584	10070930	850	45378	22.46	461.40
C37	7070669	9454489	1787	47796	25.88	517.67
C38	2120479	1679889	374	9014	18.06	549.46
C39	3979984	2782880	399	8233	10.27	302.94
C40	2120118	3301345	981	28678	47.38	889.52
C41	4046444	3071532	1760	16627	44.54	554.32
C42	4593344	2540298	1024	16118	22.83	649.72
C43	3159400	3014683	3272	48711	106.05	1654.57
C44	2333246	4324610	1955	26600	85.80	629.85

C45	3806169	1773959	1323	9963	35.59	575.10
C46	14101282	6975659	2831	120378	20.56	1767.10
C47	7964422	5803393	3575	67509	45.96	1191.19
C48	3540108	6950224	644	25532	18.63	376.17
C49	2395316	2315700	803	19092	34.33	844.25
C50	8446249	6442765	4786	61159	58.02	972.05
C51	2413547	4050377	1713	32329	72.68	817.33
C52	4159906	3354628	1575	31648	38.77	966.06
C53	5958523	10342725	1702	42969	29.25	425.42
C54	2747750	2581553	829	34578	30.89	1371.57
C55	4150195	3035605	2066	50668	50.98	1709.18
C56	7255318	5921760	5834	70244	82.34	1214.67
C57	6292843	6514873	1889	47616	30.74	748.42
C58	8407813	13211442	5620	133752	68.45	1036.69
C59	4282014	4950041	1290	16942	30.85	350.47
C60	2216103	3569704	895	21330	41.36	611.87
C61	3443205	1812525	1533	11403	45.59	644.22
C62	9031666	7983909	2131	47685	24.16	611.60
C63	4927950	8656076	1923	51015	39.96	603.50
C64	691322	1421276	91	5826	13.48	419.75
C65	3683779	3917687	2638	127658	73.33	3336.71
C66	1404263	6205522	279	10889	20.34	179.68
C67	7507379	9339214	4451	53933	60.71	591.35
C68	5336609	21706630	1259	42573	24.16	200.84
C69	2151857	1567078	596	14783	28.36	965.99
C70	2025909	5586797	863	27935	43.62	512.02
C71	4569819	4451640	13180	115320	295.34	2652.68
C72	3348334	9643018	1299	43605	39.73	463.05
C73	7664581	4845543	3728	73016	49.81	1543.03
C74	8303082	4066836	2180	38098	26.89	959.28
C75	3966585	5718294	2837	119745	73.24	2144.33
C76	3509715	2986382	486	23644	14.18	810.73
C77	5121332	8569122	1576	39398	31.51	470.80
C78	5280628	7654299	933	30136	18.09	403.16
C79	7247341	8310415	3165	78810	44.72	971.09
C80	2076429	1436955	448	14952	22.09	1065.51
C81	3134748	5975415	1644	21083	53.70	361.30
C82	4102803	2831840	1184	27816	29.55	1005.83
C83	7361977	8509806	4577	223306	63.66	2687.08
C84	9523067	6397194	5815	83227	62.53	1332.22
C85	11103038	6658310	2429	58882	22.40	905.56
C86	3903063	4445634	572	64379	15.01	1482.90
C87	5679974	4181897	4978	84932	89.74	2079.69
C88	6064567	4827020	2558	52521	43.19	1114.18
C89	2951233	4418189	2357	37881	81.78	877.96
C90	5237501	2356952	1625	20399	31.77	886.25
C91	2781574	2654606	638	18043	23.49	696.00
C92	10031561	16345626	2782	138998	28.40	870.78
C93	6395592	4180751	2106	87485	33.72	2142.79
C94	4710460	3835763	1171	29728	25.46	793.62

C95	9661562	5982124	2855	24720	30.26	423.15
C96	4264158	4362786	2655	56344	63.76	1322.46
C97	6440212	7752298	5528	111530	87.90	1473.20
C98	5648749	5740167	10502	109790	190.38	1958.57
C99	10764869	20806080	2826	90336	26.88	444.60
C100	752613	1698368	233	25469	31.70	1535.61
C101	3641916	3660181	2511	48740	70.60	1363.59
C102	7082282	6193962	7336	79053	106.07	1306.92
C103	2999799	4361786	1619	65574	55.27	1539.46
C104	4785682	7391967	5577	97065	119.33	1344.63
C105	4638718	13478364	1299	31994	28.68	243.07
C106	3481274	6036424	2217	54803	65.21	929.66
C107	6935758	9493639	5162	208500	76.21	2248.92
C108	5395068	5410454	2946	47990	55.92	908.27
C109	2099920	2803717	587	34200	28.62	1249.08
C110	2116939	3517617	2940	26517	142.21	771.93
C111	5857910	10905642	1939	107204	33.89	1006.61
C112	1854373	2888134	337	22224	18.61	787.96
C113	4692267	11794277	1815	130808	39.61	1135.70
C114	3556061	6633216	2324	60441	66.92	933.06
C115	5014083	6471212	1821	69066	37.19	1092.90
C116	2922835	3195056	1279	43248	44.81	1386.08
C117	3887261	4200868	1472	52045	38.78	1268.64
C118	5027343	9092094	1831	61071	37.29	687.81
C119	2803818	2045104	807	17389	29.47	870.68
C120	3152916	5028449	2223	63648	72.20	1296.14
C121	5550155	6622252	1635	64719	30.17	1000.75
C122	8299902	7613844	4305	86000	53.11	1156.63
C123	2317598	3114280	3525	57499	155.75	1890.61
C124	5437754	6459041	11666	153208	219.69	2428.92
C125	11562384	8522789	3895	64664	34.50	776.93
C126	1766027	3383135	284	14134	16.47	427.80
C127	10073827	8829084	28310	97986	287.77	1136.44
C128	5910509	10060797	5308	109198	91.96	1111.43
C129	5244290	3421572	3016	35162	58.89	1052.32
C130	4667706	6310702	1590	39721	34.88	644.53
C131	5315389	4441416	3036	71152	58.49	1640.46
C132	6445553	6235534	1264	48939	20.08	803.68
C133	3833148	9507971	4754	82194	127.00	885.22
C134	5399204	13875545	579	47965	10.98	353.98
C135	3827876	2526634	1738	45553	46.49	1846.18
C136	5440816	4175155	25361	63548	477.31	1558.58
C137	1257012	3511464	262	28107	21.34	819.65
C138	2419361	4456135	542	35300	22.94	811.18
C139	7660339	14594045	9174	164324	122.63	1152.99
C140	2395886	4270314	1686	26612	72.06	638.14
C141	5816349	5169664	1917	58907	33.75	1166.82
C142	3143262	6795796	1642	31854	53.49	479.98
C143	5801625	2386664	1773	21269	31.29	912.55
C144	5574908	13089130	3906	103603	71.75	810.52

C145	3220949	8748380	719	34835	22.86	407.74
C146	4988451	6043648	3766	108595	77.31	1839.97
C147	4643790	3873123	9515	79920	209.81	2112.97
C148	4089987	2058937	459	23236	11.49	1155.63
C149	6458054	3739493	4718	118429	74.81	3242.99
C150	2756644	8245966	2082	48750	77.34	605.39
C151	7361136	6659622	7984	81008	111.06	1245.60
C152	2288778	3330677	828	29355	37.04	902.50
C153	10371880	12253789	1907	88807	18.83	742.12
C154	4034010	6286701	2723	50291	69.12	819.16
C155	4406291	2103876	2472	82031	57.45	3992.62
C156	5626968	7334893	2442	64644	44.44	902.47
C157	3005978	8045317	1073	71111	36.55	905.09
C158	6303211	8994134	29978	37096	487.01	422.35
C159	5488976	3486436	1027	41777	19.16	1227.03
C160	2318299	3777266	904	19923	39.93	540.10
C161	7693492	5269302	16549	62200	220.27	1208.75
C162	5716893	10999823	5734	44949	102.71	418.44
C163	5161532	4810828	1853	22281	36.76	474.26
C164	9140195	12284422	15699	171895	175.88	1432.88
C165	1671487	1210352	1156	27936	70.82	2363.48
C166	2650403	3068726	475	16709	18.35	557.56
C167	3237880	5448687	1844	18144	58.32	340.99
C168	4293975	3348759	847	23348	20.20	713.95
C169	5496496	6619530	3289	86030	61.27	1330.83
C170	6279727	2075380	1331	34349	21.70	1694.79
C171	1804089	3555876	1049	44519	59.54	1282.03
C172	6730696	15922288	2819	83816	42.89	539.04
C173	4144817	4768197	1216	41828	30.04	898.28
C174	6664648	9353479	3681	214853	56.56	2352.17
C175	4880429	10096853	1711	109372	35.90	1109.23
C176	4081089	4330542	1597	27858	40.07	658.73
C177	3998770	4049420	2028	14357	51.93	363.05
C178	5094043	4298741	5435	19607	109.25	467.06
C179	1667465	5651823	488	35563	29.97	644.33
C180	5551514	5501435	4202	16303	77.51	303.45
C181	6002939	7035292	3611	44210	61.60	643.48
C182	413534	545269	250	12228	61.91	2296.38
C183	3272286	6977407	7344	85169	229.82	1249.94
C184	1968859	2304656	864	8433	44.94	374.69
C185	3135488	5188853	2564	27544	83.74	543.57
C186	11799537	17155550	6817	176143	59.16	1051.38
C187	844832	1410519	1683	18176	203.99	1319.53
C188	3400713	2043477	2129	15671	64.11	785.28
C189	5152970	3556392	983	34493	19.53	993.16
C190	1179571	7289586	508	26826	44.10	376.84
C191	10351010	7712350	12164	92328	120.34	1225.88
C192	6103609	3575924	12718	42700	213.37	1222.76
C193	3433715	3294073	24456	77585	729.33	2411.82
C194	2494229	5425404	975	41851	40.03	789.90

C195	9572210	8506409	3022	73220	32.33	881.42
C196	4208346	6096551	3257	69874	79.25	1173.63
C197	9733497	7577731	3829	73168	40.28	988.74
C198	5475706	6165932	4091	63554	76.50	1055.47
C199	6636704	5294796	3647	59487	56.27	1150.46
C200	4820762	9590496	1704	20617	36.20	220.13
C201	8990734	6938532	1754	38258	19.98	564.62
C202	9640802	2799113	5631	41379	59.81	1513.77
C203	5293122	9951859	3410	90967	65.97	936.01
C204	2921185	2913021	649	26766	22.75	940.89
C205	5235165	10680988	2085	37729	40.78	361.71
C206	3639278	10760612	1194	55512	33.60	528.26
C207	3819001	2348496	948	28575	25.42	1245.94
C208	2802038	2472044	1193	19275	43.60	798.43
C209	3420719	2672618	1057	31478	31.64	1206.06
C210	4597539	3520518	2818	25213	62.76	733.36
C211	7968479	3510610	1951	29229	25.07	852.57
C212	3119464	3338337	833	20532	27.34	629.80
C213	3800113	6239434	893	15846	24.06	260.06
C214	16239916	8092881	11599	83378	73.14	1054.99
C215	4953010	6709600	1386	28941	28.65	441.69
C216	11148353	10230701	7404	136345	68.01	1364.69
C217	2848392	5275407	1352	23708	48.60	460.19
C218	1481461	1512126	3717	16232	256.92	1099.22
C219	9451143	9332966	41860	180012	453.54	1975.07
C220	6170600	14477590	1388	60778	23.03	429.88
C221	4733615	3456208	2601	33209	56.27	983.91
C222	856285	2003383	331	30533	39.58	1560.65
C223	4629320	6533831	5411	90963	119.69	1425.60
C224	3367781	3737461	3044	35917	92.56	984.06
C225	5169312	5803948	962	37869	19.06	668.13
C226	15030927	9597429	6631	129890	45.17	1385.86
C227	2113994	2364271	942	27820	45.63	1204.92
C228	6768858	4609430	3387	54448	51.24	1209.58
C229	7345217	4771160	10483	41532	146.14	891.37
C230	2664471	1817238	1593	10679	61.22	601.75
C231	3120509	3085869	1800	29001	59.07	962.36
C232	3562765	4039355	1449	45940	41.65	1164.61
C233	5708336	5296355	2678	42412	48.04	820.00
C234	3987109	6216855	1086	46496	27.89	765.85
C235	3942106	3357802	2108	67933	54.76	2071.69
C236	1899767	1760285	430	11186	23.18	650.72
C237	5573884	5165475	1961	33027	36.03	654.72
C238	8476678	10772579	965	53236	11.66	506.04
C239	8630391	13529528	4821	205099	57.20	1552.32
C240	7760173	18983660	3525	73288	46.51	395.32
C241	6067441	7607346	2400	42419	40.50	570.99
C242	8123705	5214922	1130	28258	14.24	554.87
C243	3358337	6257202	984	30564	30.00	500.18
C244	9850216	9443888	6088	129625	63.29	1405.52

C245	5855304	3697789	3923	53208	68.61	1473.45
C246	2563313	6660597	2600	79266	103.87	1218.64
C247	9411485	9865736	1515	38093	16.48	395.38
C248	15125306	7644262	2430	35482	16.45	475.31
C249	2523999	2933322	1242	29190	50.39	1019.00
C250	3370652	2643490	1472	17355	44.72	672.27
C251	4395497	8497845	3390	27399	78.98	330.16
C252	9207782	11195266	798	39139	8.87	357.99
C253	5544630	3170432	1753	39343	32.37	1270.72
C254	7085272	3876414	2038	38760	29.45	1023.89
C255	9646556	9837737	10069	138814	106.88	1444.90
C256	4343544	3868044	1283	24022	30.25	635.94
C257	5143968	8346967	1706	81991	33.96	1005.86
C258	12057934	5954084	12680	38672	107.68	665.09
C259	2903899	5729322	3753	25592	132.34	457.41
C260	2350427	3939612	851	43374	37.08	1127.39
C261	14035222	3617796	9846	25444	71.84	720.18
C262	5238304	8800976	4206	87176	82.22	1014.30
C263	6389488	5713865	1528	15130	24.49	271.15
C264	5050987	4828480	1962	55087	39.78	1168.26
C265	6639315	11543292	7768	60744	119.81	538.86
C266	3797930	11327108	3390	96866	91.40	875.69
C267	5672356	5907952	5597	63812	101.04	1106.03
C268	8047107	5355918	6158	37695	78.36	720.69
C269	3264603	8788133	2313	40129	72.55	467.59
C270	5595968	5589832	9177	48089	167.93	880.94
C271	5306083	6114829	4220	35373	81.44	592.36
C272	2071426	3025229	548	33474	27.09	1133.05
C273	4204101	2544518	611	8833	14.88	355.47
C274	2706617	1576260	1014	13554	38.36	880.52
C275	3765283	6216775	747	21667	20.32	356.89
C276	6322634	7087146	14502	308112	234.87	4451.82
C277	7194030	8384521	1623	45853	23.10	560.00
C278	1556777	4063853	3695	96188	243.05	2423.72
C279	5475717	7333630	1541	28205	28.82	393.83
C280	2600253	4560864	778	19701	30.64	442.32
C281	2629236	5773446	2640	56772	102.82	1006.93
C282	3185472	7903857	602	22433	19.35	290.64
C283	3189001	3055707	1260	25741	40.46	862.61
C284	1610256	1639303	2193	15584	139.46	973.46
C285	3881466	3440700	823	22756	21.71	677.25
C286	3097041	4155568	619	7380	20.47	181.86
C287	7103260	7092993	2759	69651	39.77	1005.54
C288	7128992	4381800	3748	41163	53.84	961.95
C289	3547618	6069220	3009	135786	86.85	2290.98

Supplementary Table 2. **Disease-free survival (DFS) by intratumoral (iTIL) or stromal (sTIL) tumor-infiltrating lymphocyte density quartiles**

	Disease-free survival		
	**5-year DFS rate (%)**	**HR (95% CI)**	**p-value**
sTIL			
<25%	78.9	Reference	-
25–50%	87.5	0.66 (0.31–1.43)	0.293
50–75%	94.3	0.24 (0.08–0.72)	0.010
≥75%	97.1	0.23 (0.08–0.69)	0.009
iTIL			
<25%	82.1	Reference	-
25–50%	94.1	0.28 (0.09–0.85)	0.025
50–75%	87.4	0.89 (0.42–1.89)	0.757
≥75%	94.3	0.28 (0.09–0.85)	0.025

Supplementary Table 3. Comparison of time to recurrence (TTR) and disease-free survival (DFS) by the combined iTIL/sTIL risk groups

	**Right-sided**		**Left-sided**	
	**5-year recurrence-free rate (%)**	**p-value**	**5-year recurrence-free rate (%)**	**p-value**
High-risk	58.3%	-	78.3%	-
Intermediate-risk	87.8%	0.116	88.5%	0.134
Low-risk	95.6%	0.011	96.8%	0.002
	**Stage II**		**Stage III**	
	**5-year recurrence-free rate (%)**	**p-value**	**5-year recurrence-free rate (%)**	**p-value**
High-risk	66.7%	-	76.8%	-
Intermediate-risk	94.7%	0.057	84.9%	0.208
Low-risk	100%	0.933	93.8%	0.008

	**Right-sided**		**Left-sided**	
	**5-year DFS rate (%)**	**p-value**	**5-year DFS rate (%)**	**p-value**
High-risk	50.0%	-	78.3%	-
Intermediate-risk	87.8%	0.115	86.1%	0.235
Low-risk	93.3%	0.024	96.8%	0.002
	**Stage II**		**Stage III**	
	**5-year DFS rate (%)**	**p-value**	**5-year DFS rate (%)**	**p-value**
High-risk	66.7%	-	72.7%	-
Intermediate-risk	94.7%	0.063	82.4%	0.218
Low-risk	100%	0.008	92.4%	0.004

Supplementary Fig.1. **Correlations between the stromal tumor-infiltrating lymphocyte (sTIL) density and the TIL scores estimated by the International TILs Working Group (ITWG) guideline adapted for colon cancer**

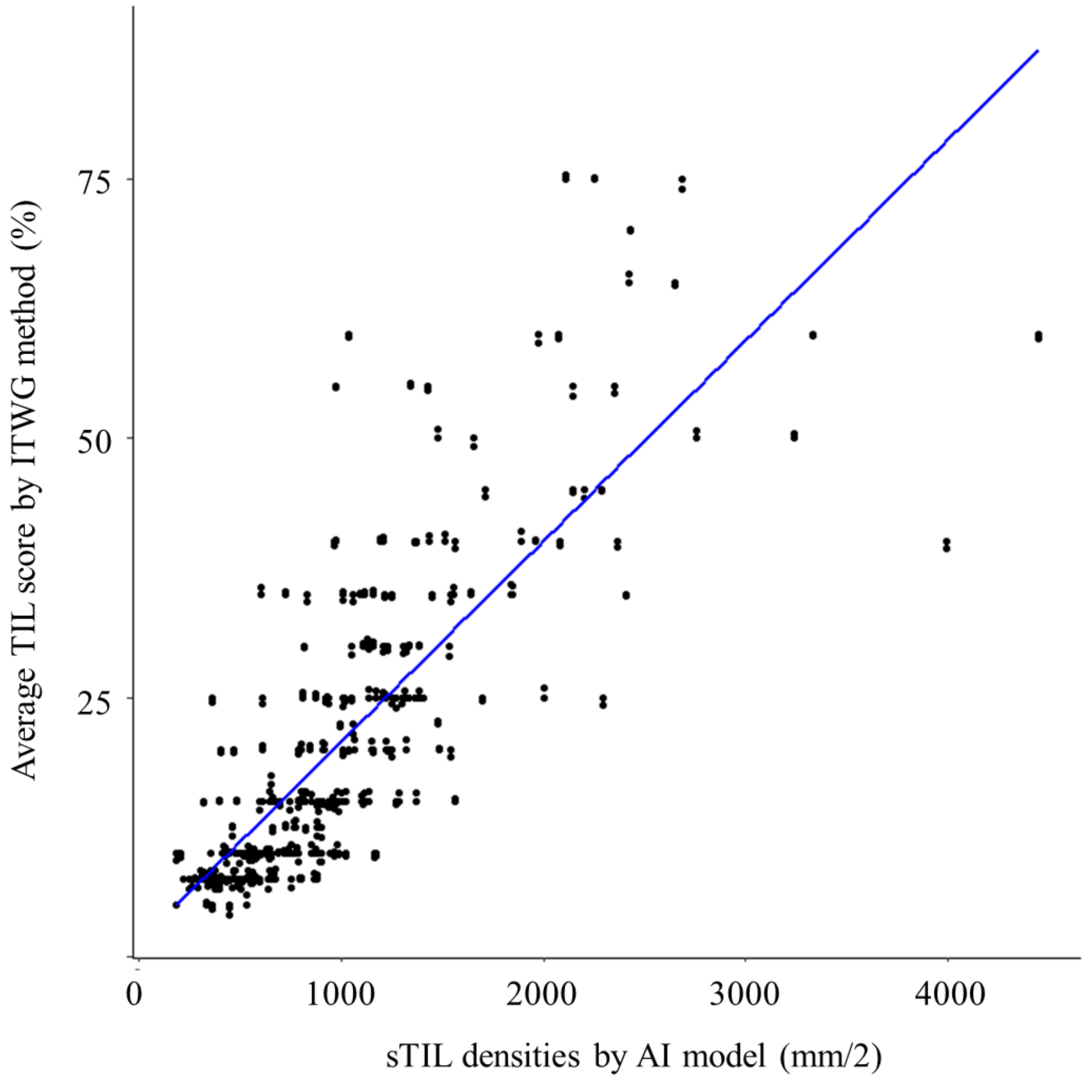

Supplementary Fig.2. **Kaplan Meier curves of disease-free survival (DFS) according to the combined intratumoral (iTIL)/stromal (sTIL) tumor-infiltrating lymphocyte risk groups (HR, hazard ratio; CI, confidence interval)**

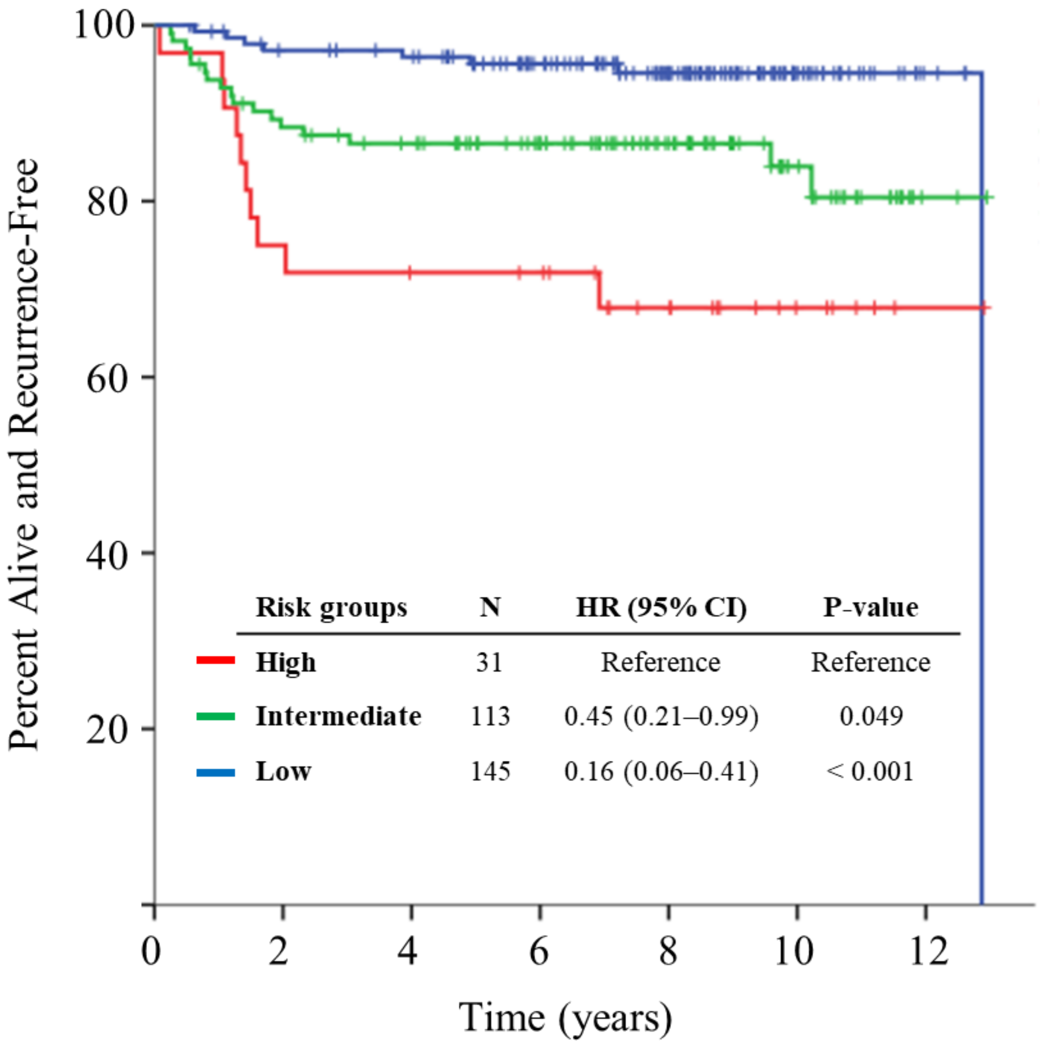

Supplementary Fig.3. **Schematic workflow of Lunit SCOPE IO**

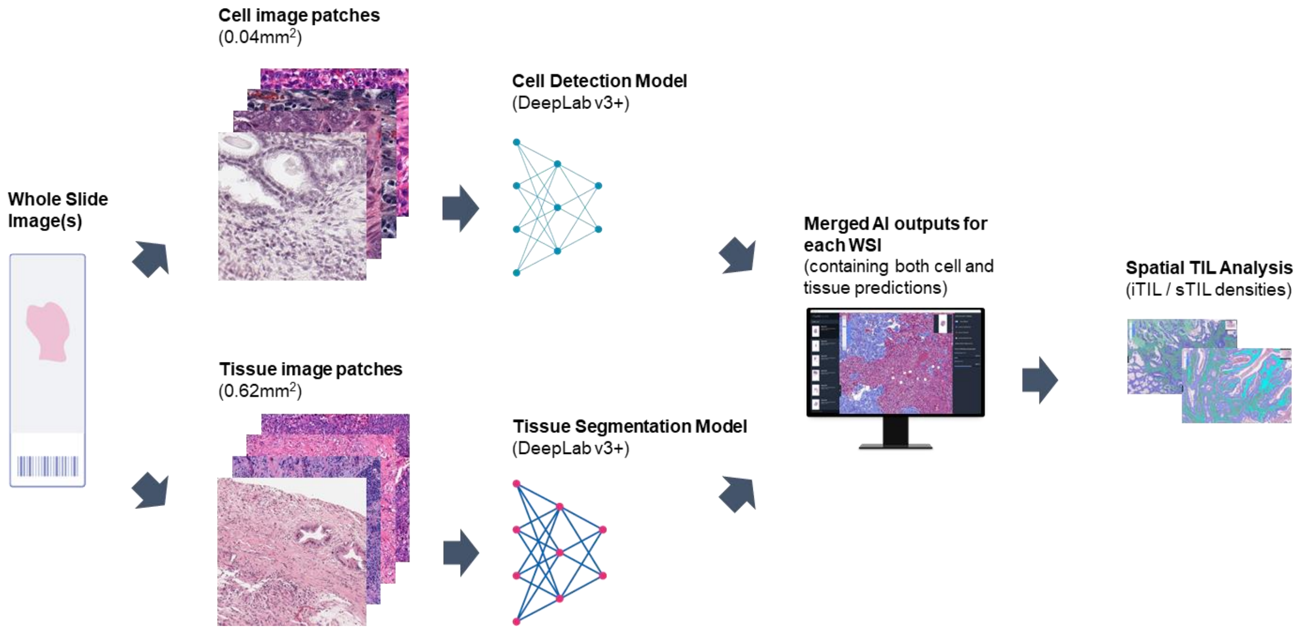

Supplement: Supplementary file 1 — Supplementary Tables 1-3, Supplemnetary Figures 1-3 [file 41698_2023_470_MOESM1_ESM.pdf]
